# Pathophysiology of Acute Coronary Syndromes—Diagnostic and Treatment Considerations

**DOI:** 10.3390/life13071543

**Published:** 2023-07-12

**Authors:** Panagiotis Theofilis, Evangelos Oikonomou, Christos Chasikidis, Konstantinos Tsioufis, Dimitris Tousoulis

**Affiliations:** 1 First Department of Cardiology, “Hippokration” General Hospital, University of Athens Medical School, 115 27 Athens, Greece; ptheofilis@med.uoa.gr (P.T.); ktsioufis@med.uoa.gr (K.T.); 2 Third Department of Cardiology, Thoracic Diseases General Hospital “Sotiria”, University of Athens Medical School, 115 27 Athens, Greece; boikono@med.uoa.gr; 3 Department of Cardiology, General Hospital of Corinth, 201 00 Corinth, Greece; chasikid@otenet.gr

**Keywords:** pathophysiology, acute coronary syndromes, plaque erosion, plaque rupture, calcified nodule

## Abstract

Coronary artery disease and acute coronary syndromes are accountable for significant morbidity and mortality, despite the preventive measures and technological advancements in their management. Thus, it is mandatory to further explore the pathophysiology in order to provide tailored and more effective therapies, since acute coronary syndrome pathogenesis is more varied than previously assumed. It consists of plaque rupture, plaque erosion, and calcified nodules. The advancement of vascular imaging tools has been critical in this regard, redefining the epidemiology of each mechanism. When it comes to acute coronary syndrome management, the presence of ruptured plaques almost always necessitates emergent reperfusion, whereas the presence of plaque erosions may indicate the possibility of conservative management with potent antiplatelet and anti-atherosclerotic medications. Calcified nodules, on the other hand, are an uncommon phenomenon that has largely gone unexplored in terms of the best management plan. Future studies should further establish the importance of detecting the underlying mechanism and the role of various treatment plans in each of these distinct entities.

## 1. Introduction

Coronary artery disease (CAD) and acute coronary syndromes (ACS) represent a significant public health burden worldwide, contributing to substantial morbidity and mortality rates. Epidemiological studies have highlighted the widespread prevalence and considerable impact of these conditions on individuals and healthcare systems. CAD refers to the narrowing or blockage of the coronary arteries, which supply oxygen and nutrients to the heart muscle. It is the leading cause of death globally and is responsible for a considerable proportion of cardiovascular-related deaths. ACS encompasses a spectrum of clinical presentations, ranging from unstable angina to myocardial infarction (MI), the most severe form. The main pathophysiologic mechanisms underlying the development of ACS involve plaque rupture, erosion, and calcified nodules [1,2]. In this review article, we delve into those distinct entities, highlighting their epidemiology, risk factors, diagnostic and histologic findings, as well as the available therapeutic approaches.

## 2. Brief Overview of Atherosclerosis Pathophysiology

The atherosclerosis cascade has long been considered a multistep process. Based on numerous reports from experimental studies, it is now established that the presence of endothelial dysfunction is essential for the initiation of atherosclerosis, since it facilitates the uptake of low-density lipoprotein (LDL) in the subendothelial space with the assistance of proteoglycans located in the extracellular matrix [3,4]. Subsequently, LDL modification and mainly oxidation ensues [3,4]. Established cardiovascular risk factors are implicated in the progression of endothelial dysfunction [3]. Following endothelial activation, various molecules (selectins, adhesion molecules) enable leukocyte trafficking and penetration in the subintimal space [5]. Those leukocytes are then differentiated into macrophages and engulf oxidized LDL [6]. Consequently, noxious inflammatory and oxidative responses arise [7,8], together with the activation and proliferation of vascular smooth muscle cells (VSMCs) in the media layer [9]. VSMCs in particular may also display phagocytic actions through the uptake of oxidized LDL, being major contributors in atherosclerotic plaques [10,11].

## 3. Plaque Rupture

Plaque rupture originates within a susceptible plaque, which has a thin fibrous cap covering a lipid-rich core (Table 1). A pool of inflammatory cells, including macrophages and T lymphocytes, lies under the fibrous cap and is engaged in continuing plaque inflammation and remodeling. The rupture of the fibrous cap exposes the lipid-rich core to the bloodstream, resulting in a chain reaction of events. Mechanical stress, inflammation, and extracellular matrix degradation are all factors that lead to plaque rupture. Hemodynamic factors such as shear stress and cyclic strain put mechanical stress on the plaque, which can lead to cap thinning and, ultimately, rupture. Furthermore, prolonged plaque inflammation stimulates the release of proteolytic enzymes, specifically matrix metalloproteinases (MMPs), which degrade extracellular matrix components. A mismatch between MMPs and their inhibitors weakens the fibrous cap, making it more prone to rupture. Inflammation is important in both the beginning and development of plaque rupture. Circulating monocytes cling to active endothelial cells and move into the intima, where they differentiate into macrophages. Internalizing oxidized low-density lipoproteins, these macrophages change into foam cells and contribute to the formation of the lipid-rich core. Inflammatory cytokines, such as interleukin-6 (IL-6) and tumor necrosis factor-alpha (TNF-α), promote plaque vulnerability by perpetuating inflammation. Furthermore, local inflammatory mediators, including C-reactive protein (CRP), play an important role in plaque destabilization, increasing the risk of rupture.

### 3.1. Epidemiology and Risk Factors

Plaque rupture is a critical mechanism of coronary thrombosis, accounting for up to 75% of all ACS. Traditional cardiovascular risk factors, such as hypertension, smoking, diabetes, hyperlipidemia, and obesity, as well as non-traditional risk factors, such as inflammation, infection, and hereditary vulnerability, all contribute to the formation of susceptible plaques. For example, hypertension exerts mechanical stress on the arterial wall, which can hasten plaque growth and increase the risk of rupture. Diabetes and smoking can aggravate plaque inflammation, compromise endothelial function, and contribute to plaque instability.

### 3.2. Diagnosis of Rupture-Prone Coronary Atherosclerotic Plaques

Several histological traits are linked to susceptible plaques that are prone to rupture. Thin fibrous caps (<65 μm) have been noted as an important predictor of plaque vulnerability. Large lipid cores, increased macrophage density, and neovascularization all contribute to plaque instability. Plaque susceptibility is further modulated by the presence of intraplaque hemorrhage and calcification, with hemorrhage stimulating inflammation and calcification potentially stabilizing the plaque. Thrombi on ruptured plaques are rich in fibrin with a significantly higher expression of tissue factor and CRP.

Plaque rupture diagnosis in clinical practice remains difficult. Intravascular imaging methods such as intravascular ultrasonography (IVUS) and optical coherence tomography (OCT) give excellent insights into plaque shape and rupture features. OCT and IVUS remain the gold standard for assessing vulnerable coronary atherosclerotic plaques that are prone to rupture. These two techniques provide complimentary data on atherosclerotic plaques, since OCT can better characterize tissue types and the presence of thin cap fibroatheromas (TCFAs) based on a very high resolution, while IVUS has a greater penetration depth and can provides an adequate assessment of plaque burden and vessel remodeling. The latter is an important feature of culprit lesions in ACS, which are also characterized by a large plaque burden [12]. We should also state that OCT and IVUS may additionally identify healed plaque ruptures that were asymptomatic, indicating that plaque rupture is a phenomenon occurring more frequently than clinically expected [12,13].

Still, their invasive nature necessitates the development of non-invasive modalities, such as blood-based biomarkers and imaging methods, that can quickly and adequately evaluate the existence of high-risk features for rupture-prone plaques and ultimately contribute to the risk prognostication of such patients. Published research on rupture-prone plaques has found a strong association between non-invasive and invasive imaging modalities, as well as plaque histology. Furthermore, changes in circulating inflammatory and extracellular matrix degradation indicators may suggest an additional risk factor. However, the scarcity of large-scale, multicenter randomized clinical studies and registries is a barrier to the widespread use of these modalities in clinical practice.

Starting with the best studied inflammatory measure, CRP, high levels have been linked to the existence and load of TCFAs in ACS patients [14,15,16], as well as with plaque rupture [17]. With an area under the receiver operating characteristic curve (AUROC) of 0.83 and an optimum cutoff of 9.9 ng/mL, MMP-9 has been related to the presence of TCFAs in the culprit lesion of patients with an ACS [18]. Furthermore, microRNAs are being studied in the treatment of atherosclerotic diseases [19,20], with initial results linking their levels to susceptible plaque features [21].

Moving to non-invasive imaging, plaques identified as TCFAs in IVUS had a heterogeneous shape in multi-slice computed tomography (CT) [22]. Small patchy plaque calcifications detected via coronary CT angiography (CCTA) were likewise linked to the proportion of necrotic core and the frequency of TCFA as determined by IVUS [23]. When compared to non-TCFA culprit lesions, OCT-detected TCFAs exhibited a higher degree of positive remodeling and a lower attenuation value [24]. However, a ring-like enhancement on CT was prevalent in TCFA, with poor diagnostic acuity (sensitivity: 44%, specificity: 96%) [24]. Ito et al. found that an attenuation value of 62.4 Hounsfield units (HU), a remodeling index of 1.08, and a signet ring-like enhancement were independent predictors of OCT-defined TCFA in a multivariate analysis of coronary atherosclerotic plaques in 81 patients with clinically suspected CAD [25]. IVUS-derived TCFA may be associated with a high necrotic core/fibrous plaque ratio [26]. In addition, increased epicardial fat content and density have been identified as an independent predictor of TCFAs [27,28]. Another non-invasive way of assessing atherosclerotic plaques is dual-source CT (DSCT). A low-attenuation plaque volume bigger than 8 mm^2^ generated from DSCT demonstrated a remarkable diagnostic capability for IVUS-defined TCFA, with 91%, 84.6%, and 96.8% accuracy, sensitivity, and specificity, respectively [29]. Other methods have been also studied, albeit to a lesser degree [30]. The increased use of magnetic resonance imaging in cardiovascular disorders has resulted in significant advances in the identification of susceptible plaque. At the same time, positron emission tomography investigations may confront limitations in terms of myocardial uptake and motion correction, which should be addressed.

### 3.3. Clinical Implications

Plaque rupture is a crucial step in the progression of ACS. When a susceptible plaque ruptures, the thrombogenic lipid-rich core is exposed to the circulation, resulting in platelet activation and aggregation. This causes an occlusive thrombus to develop, resulting in myocardial ischemia and possible myocardial infarction. Depending on the level of myocardial damage, the clinical appearance of ACS can range from unstable angina (transient ischemia without myocardial injury) to non-ST-segment elevation myocardial infarction (NSTEMI) or, most commonly, ST-segment elevation myocardial infarction (STEMI).

Management strategies for ACS with plaque rupture uniformly focus on restoring blood flow to the affected coronary artery promptly. This almost always involves a combination of antiplatelet agents, anticoagulants, and revascularization procedures, such as percutaneous coronary intervention (PCI) or coronary artery bypass grafting (CABG). Additionally, secondary prevention measures, including lifestyle modifications, statin therapy, and cardiac rehabilitation, are crucial in reducing the risk of recurrent events. 

## 4. Plaque Erosion

Even though plaque rupture is the most well-defined underlying mechanism of ACS, an alternative process known as plaque erosion has gained recognition as a distinct etiology. Plaque erosion refers to the thrombotic occlusion of a coronary artery without the disruption of the fibrous cap near an area of endothelial denudation.

### 4.1. Epidemiology

The presence of plaque erosion in culprit coronary arteries of patients with an ACS has been increasingly more common over the years. Two main factors could be responsible for this observation. First and foremost, although early studies have noted a plaque erosion prevalence in approximately 20% of ACS patients, they were based on the autopsy studies of fatal acute cardiovascular events that may be more commonly associated with plaque rupture [31]. Invasive vascular imaging was not widely used; thus, these rates could have been falsely low. Moreover, the increased awareness regarding the management of major cardiovascular risk factors may have accounted for the attenuation of atherosclerosis progression and the stabilization of atherosclerotic plaques [31]. Specifically, aggressive lipid-lowering therapies (statins, ezetimibe, bempedoic acid, and proprotein convertase subtilisin/kexin type 9 (PCSK9) inhibitors, including inclisiran) [32,33,34,35,36], novel antidiabetic agents with pleiotropic effects (sodium–glucose cotransporter-2 inhibitors, glucagon-like peptide-1 receptor agonists) [37,38], and contemporary antihypertensive medications (renin–angiotensin–aldosterone system blockers) are common approaches with significant anti-atherosclerotic effects. Smoking cessation and adaptation of a healthier lifestyle in terms of diet and exercise could be essential determinants of atherosclerosis stabilization [39,40,41]. Moreover, the importance of inflammation should be also stressed, as the residual inflammatory risk is a crucial aspect of the atherosclerotic process that could be medically managed with agents such as colchicine [42]. As a result, the regression of atherosclerosis could prevent vulnerable, rupture-prone plaque formation and lead to more cases of NSTEMI with plaque erosion. 

We should also mention that the use of invasive vascular imaging methods and the establishment of possible plaque erosion criteria could have contributed to a greater degree of plaque erosion detection. It is, therefore, unsurprising that contemporary studies in the field have produced plaque erosion rates of approximately 40% [31]. However, even such rates may not reflect the true prevalence of plaque erosion, since only a limited proportion of patients with ACS undergo invasive coronary imaging with OCT or IVUS.

### 4.2. Pathogenesis and Risk Factors

The pathogenesis of plaque erosion involves a combination of factors, including endothelial dysfunction, inflammation, and platelet activation. Endothelial dysfunction, characterized by impaired nitric oxide bioavailability and the increased expression of adhesion molecules, leads to a pro-thrombotic state. Inflammatory processes, driven by infiltrating immune cells and activated endothelial cells, further contribute to plaque instability. Underlying systemic conditions, such as hypertension, hyperlipidemia, and diabetes, play a role in promoting endothelial dysfunction and inflammation, thus increasing the risk of plaque erosion. Two steps may be involved in the pathophysiology of plaque erosion. Local flow perturbation disrupted endothelial shear stress (ESS), low-level innate immune activation of endothelial cells (via Toll-like receptor 2 activation), and endothelial cell desquamation or apoptosis (mediated in part by Toll-like receptor 2, matrix metalloproteinase 2, CD8+ T lymphocytes, and myeloperoxidase pathways) are all examples of the first step. In the second step, activated endothelial cells produce chemokines, such as interleukin-8, which recruit leukocytes and promote the creation of neutrophil extracellular traps (NETs), which, in turn, exacerbate endothelial damage and lead to local thrombus formation.

ACS patients with plaque erosion are most commonly younger than those presenting with plaque rupture [43]. Moreover, although early studies suggested female sex as a risk factor for plaque erosion, recent in vivo OCT studies have shown similar rates of plaque erosion across the sexes [43,44]. No ethnic or race disparities have been noted [45]. Concerning traditional cardiovascular risk factors, these may be encountered less frequently in ACS patients with plaque erosion. Notably, in one study, the absence of diabetes mellitus and normal renal function were independent predictors of plaque erosion [46]. Compared to plaque rupture, plaque erosion is also associated with a more favorable lipid profile [47].

### 4.3. Diagnosis

Histologically, eroded plaques exhibit distinct features that differentiate them from ruptured plaques. Eroded plaques often have a thick fibrous cap without evidence of cap disruption. Instead of a lipid core, they commonly demonstrate superficial platelet-rich (white) thrombi adherent to an intact endothelial surface. Eroded plaques were rich in extracellular matrix components, such as proteoglycans and glycosaminoglycans, rather than lipids. Eroded lesions had fewer inflammatory cells and more vascular smooth muscle cells than ruptured plaques. The absence of a fibrous cap rupture and the presence of adherent thrombi are key histopathological characteristics distinguishing plaque erosion from plaque rupture.

Intravascular imaging is perhaps the method of choice in identifying plaque erosion, namely with OCT, as it has a 10-fold higher spatial resolution than IVUS. OCT is unable to view the endothelium monolayer directly. Because endothelial cell loss is a pathological characteristic of plaque erosion, this constraint prevents the use of endothelial cell absence as an OCT criterion for identifying plaque erosion. OCT has developed a plaque classification algorithm as a result of this. In patients with ACS, the absence of fibrous cap rupture at the culprit lesion on OCT implies plaque erosion, according to this strategy. The identification of a thrombus with an intact underlying plaque is required for a definitive diagnosis of plaque erosion. If the luminal surface of the plaque appears sloppy in the absence of a thrombus or if there is a thrombus that hinders the visibility of an underlying plaque without a subjacent lipid accumulation or calcium proximal or distal to the lesion, plaque erosion is suspected.

### 4.4. Clinical and Therapeutic Implications

Eroded plaques are less likely to cause ST-segment elevation myocardial infarction (STEMI) but are frequently implicated in cases of non-ST-segment elevation myocardial infarction (NSTEMI) [46]. In terms of angiographic properties, plaque erosion is associated with a less complicated and widespread atherosclerotic pattern than plaque rupture [48,49,50]. A number of studies have found that plaque erosion is preferentially localized in the left anterior descending artery (LAD), notably in the proximal and middle segments, which are grouped around a bifurcation [48,49,50]. This usual distribution may represent distinct local hemodynamic pressures working within the coronary circulation, such as altered endothelial shear stress, which may be more prominent in a conduit with side branches, such as the LAD [51]. Patients who have erosion have a lower total atherosclerotic disease burden than those who have plaque rupture, as evidenced by less frequent multivessel disease involvement, a lower Syntax score, and a lower Gensini score [48]. These characteristics are consistent with OCT evidence revealing decreased pancoronary vulnerability in patients with plaque erosion and may help to explain why these individuals have a better prognosis than those with plaque rupture [52,53]. Plaque erosion lesions are less commonly related to a baseline thrombolysis in myocardial infarction (TIMI) flow grade 1. Furthermore, plaque erosion is frequently linked with a simpler angiographic lesion phenotype. Plaque erosion generally exhibits a more “simple” A/B1 lesion phenotype when using the ACC/AHA lesion categorization, as opposed to plaque rupture, which typically exhibits a “complex” B2/C lesion phenotype [54]. Plaque erosion is more commonly linked with a concentric type I lesion, whereas plaque rupture is associated with an eccentric type II lesion, according to the Ambrose lesion classification [54]. Plaque erosion, unlike plaque rupture, is less usually associated with angiographic signs of calcification and thrombus [54].

Patients with plaque erosion often have a better prognosis and a lower risk of adverse cardiovascular events compared to those with plaque rupture. Since clinical studies showed favorable outcomes in ACS patients with plaque erosion versus rupture and thrombi associated with eroded plaques are platelet-rich, it is plausible that a less invasive management strategy (antithrombotic therapy without stenting) may be safe and effective in these patients. In the EROSION trial, 60 ACS patients with plaque erosion diagnosed by OCT (96.7% with STEMI), residual diameter stenosis <70%, and thrombolysis in myocardial infarction flow grade 3 on angiography were treated with antithrombotic therapy alone without stent implantation [55]. The use of a glycoprotein IIb/IIIa inhibitor (tirofiban) and manual aspiration thrombectomy was significant (63% and 85%, respectively). After one month, all patients had a repeat coronary angiography and OCT. At 1 month, 78% of these patients attained the main goal of a >50% decrease in thrombus volume. A one-year follow-up study revealed a substantial decrease in median thrombus volume from one month to one year, with 92.5% of patients free of serious adverse cardiovascular events [56]. These findings implied that antithrombotic treatment without stenting may be an option for selected individuals with ACS caused by plaque erosion. However, according to the EROSION study’s 4-year follow-up analysis, the incidence of MACE increased to 23%, primarily due to nonurgent target lesion revascularization that was not associated with death, heart failure, stroke, recurrent myocardial infarction, unstable angina-induced rehospitalization, or coronary artery bypass grafting [57]. In an observational analysis of 232 patients, including a fraction from the initial EROSION trial, Yin and colleagues expanded their understanding of this field [58]. During a median follow-up of 2.9 years, 50 of 232 patients (21.6%) developed MACE (six patients died, three patients had nonfatal reinfarction, twenty-nine patients had target lesion revascularization, thirty-six patients had angina pectoris rehospitalization, two patients had severe bleeding, and five patients had a stroke). MACE was predicted by age, percentage of area stenosis, and thrombus load, according to multivariate Cox regression analysis. The threshold values for predictors were age ≥60 years and the percentages of area stenosis and thrombus load were ≥63.5% and ≥18.5%, respectively, and when all three of these values were present, the risk of MACE spiked to a very high level of 58%.

Finally, another open question is whether balloon angioplasty alone, or drug-coated balloon angioplasty, might be effective for plaque erosion; such an approach might still avoid a stent-based strategy for the majority of patients, but provide a lower area of stenosis, a driver of future MACE.

## 5. Calcified Nodules

The role of calcified nodules in ACS has gained increasing attention. Calcified nodules refer to discrete calcified structures within the coronary arteries that can contribute to luminal narrowing and subsequent ischemic events. The formation of calcified nodules in coronary arteries involves a complex interplay of biological processes, including inflammation, osteogenic transformation, and matrix remodeling. Chronic inflammation within atherosclerotic plaques triggers the release of pro-inflammatory cytokines and growth factors, stimulating the migration and differentiation of vascular smooth muscle cells (VSMCs) into osteoblast-like cells. These VSMCs promote the deposition of calcium phosphate crystals, leading to the development of calcified nodules. Matrix remodeling, characterized by the altered expression of matrix metalloproteinases and their inhibitors, further contributes to the process.

### 5.1. Epidemiology and Risk Factors

Calcified nodules are responsible for 5.3% of ACS [59]. Several traditional risk factors for atherosclerosis, including advanced age, male gender, hypertension, dyslipidemia, and diabetes mellitus, are associated with an increased likelihood of calcified nodules [60]. In addition, emerging risk factors, such as chronic kidney disease and genetic predisposition, have been implicated [60]. These risk factors promote vascular calcification by disrupting the balance between calcification inhibitors and promoters, favoring the deposition of calcium within the arterial wall.

### 5.2. Histology and Diagnostic Modalities 

A recent study provided additional insight into calcified nodules [60]. Histologically, all calcified nodules have a luminal fibrin/platelet thrombus, with several nodular shards of calcification piercing the overlaying fibrous cap and emerging into the luminal space, as well as indications of endothelial cell loss. The underlying plaques of the eruptive calcified nodules are mostly eccentric, with nonocclusive thrombi predominating. The fractured nodules frequently disturb the underlying medial wall as well. The percentage of intimal area occupied by calcification is 44.4% on average. Necrotic cores are uncommon. Immunohistochemical staining has shown that fibrin is abundant around eruptive calcified nodules, with fewer platelet aggregations interspersed.

IVUS is an important imaging modality in the diagnosis of calcified nodules. Five types have been defined, namely Type 1: an eccentric calcified nodule without calcification at the opposite site of calcified nodule; Type 2: an eccentric calcified nodule with broad (≥180° arc) superficial calcification at the opposite site of calcified nodule; Type 3: an eccentric calcified nodule with narrow (<180° arc) superficial calcification pattern at the opposite site of calcified nodule; Type 4: multiple calcified nodules within the lumen; and Type 5: calcified nodule with visible luminal thrombus [61]. OCT is another method with the ability to discriminate calcified nodules, which are present when fibrous cap disruption is detected over a calcified plaque characterized by protruding calcification, superficial calcium, and the presence of substantive calcium proximal and/or distal to the lesion [43].

### 5.3. Clinical Implications

Calcified nodules have been implicated in the pathogenesis of ACS, particularly in patients with non-obstructive coronary artery disease. They contribute to luminal narrowing and impaired coronary blood flow, leading to myocardial ischemia and the development of unstable angina or myocardial infarction. Calcified nodules can also serve as a source of emboli, promoting microvascular obstruction and adverse clinical outcomes.

The presence of calcified nodules is associated with a higher risk of adverse cardiovascular events, including target vessel revascularization, recurrent ACS, and mortality. This observation could be related to the continuous growth and protrusion of the calcified mass [59]. Surprisingly, rather than neointimal hyperplasia, 78% of in-stent restenosis at calcified nodules lesions was caused by the creation of a projecting mass with acoustic shadowing. These data imply that even after stent insertion, the calcified nodules continue to protrude. In-stent restenosis at calcified nodules lesions has been described in one pathohistological investigation as calcified nodules protrusion through the stent struts and thrombus or neointima calcification within the implanted stent [62]. According to IVUS imaging findings, the former mechanism appears to be more likely to explain an elevated target lesion revascularization rate [59]. Given that drug-eluting stent effectiveness is mostly determined by the prevention of neointimal hyperplasia, calcified nodules lesions may not react well to newer-generation DES implantation.

According to Prati et al., the presence of calcified nodules in non-culprit coronary plaques was also related with poorer clinical outcomes, such as cardiac mortality and target-vessel ACS [63]. As a result, calcified lesions containing calcified nodules might be classified as high-risk lesions. There is no validated therapy for enhancing clinical outcomes after PCI in calcified lesions with calcified nodules or preventing calcified nodule-related future coronary events; nonetheless, the appropriate identification of calcified nodules is critical for the risk stratification of coronary events.

## 6. Conclusions

Acute coronary syndromes have been at the forefront of scientific research due to their presumed healthcare burden. Recent evidence suggests that their pathophysiology is more diverse than previously thought, involving plaque rupture, plaque erosion, and calcified nodules. These distinct mechanisms are characterized by specific histologic features and different clinical outcomes. The evolution of vascular imaging techniques has been pivotal in this field, redefining the epidemiology of each mechanism. Concerning acute coronary syndrome management, the identification of ruptured plaques almost universally necessitates emergent reperfusion, while the presence of plaque erosions may suggest the possibility of conservative management with intensified antiplatelet and anti-atherosclerotic medications. On the other hand, calcified nodules represent rarer entities that remain largely unexplored regarding their optimal management strategy. It becomes clear that, although the management of acute coronary syndromes has significantly progressed through the years, the identification of the specific underlying mechanism opens a path for personalized treatment decisions. Therefore, future studies should further establish the importance of detecting the underlying mechanism and the role of various available treatment plans in each of these distinct entities.

## Figures and Tables

**Table 1:** Differences in thrombosis due to atherosclerotic plaque rupture or erosion.

	Erosion	Rupture
Fibrous cap	Thick and intact	Thin, fissured
Thrombus	“White” Rich in platelets	“Red” Rich in fibrin
Mechanism of activation	Collagen	Tissue factor
Prevailing cells	Smooth muscle cells	Macrophages
Remodeling	Lower degree, outward remodeling	Marked positive remodeling
NET presence	++	+/±
Common clinical outcome	Non-STEMI	STEMI

NET: neutrophil extracellular trap; STEMI: ST-segment elevation myocardial infarction.

## Data Availability

Not applicable.
